# Using Sediment Bacterial Communities to Predict Trace Metal Pollution Risk in Coastal Environment Management: Feasibility, Reliability, and Practicability

**DOI:** 10.3390/toxics12120839

**Published:** 2024-11-22

**Authors:** Yuanfen Xia, Jiayuan Liu, Xuechun Yang, Xiaofeng Ling, Yan Fang, Zhen Xu, Fude Liu

**Affiliations:** 1 State Power Environmental Protection Research Institute, Nanjing 210031, China; wzwfenfen@163.com (Y.X.); xygslxf@163.com (X.L.); wyl20180227@sina.com (Y.F.); alex_xuzhen@163.com (Z.X.); 2 School of Environmental Science and Safety Engineering, Tianjin University of Technology, Tianjin 300384, China; yxcxcm@163.com

**Keywords:** accumulation risk, bacterial community, coastal watershed, tidal gate, trace metals

## Abstract

The distribution of trace metals (TMs) in a continuous water body often exhibits watershed attributes, but the tidal gates of the coastal rivers may alter their transformation and accumulation patterns. Therefore, a tidal gate-controlled coastal river was selected to test the distribution and accumulation risks of Al, As, Cr, Cu, Fe, Mn, Ni, Sr, and Zn in the catchment area (CA), estuarine area (EA), and offshore area (OA). Associations between TMs and bacterial communities were analyzed to assess the feasibility of using bacterial parameters as ecological indicators. The results showed that As and Cr were the key pollutants due to the higher enrichment factor and geoaccumulation index, reaching slight to moderate pollution levels. The Nemero index was highest in EAs (14.93), indicating a higher pollution risk in sediments near tide gates. Although the TM dynamics can be explained by the metal-indicating effects of Fe and Mn, they have no linear relationships with toxic metals. Interestingly, the metabolic abundance of bacterial communities showed good correlations with different TMs in the sediment. These results highlight bacterial community characteristics as effective biomarkers for assessing TM pollution and practical tools for managing pollution control in coastal environment.

## 1. Introduction

At the global scale, trace metal (TM) pollution poses a significant threat to coastal ecosystems [1,2,3,4]. TMs are generally derived from natural (erosion and mineral weathering) and anthropogenic (industrial or mining activities) sources and are transferred to the coastal ecosystems through river transport and wastewater discharge [1,2,5,6]. Given their toxic, persistent, and non-releasable properties, TMs in sediments can persist in an elemental state, which disrupts food chains and threaten ecological balance [7]. Even when TM concentrations fall within regional environmental standards, their latent risks to humans and ecosystems health remain severe and require urgent attention [8,9].

TM pollution has captured the attention of many environmental scientists, due to its ability to induce bioaccumulation and disrupt the delicate ecological equilibrium, particularly in coastal river ecosystems [8,10]. TMs not only migrate over long-distances but are also influenced by the migration behavior of aquatic organisms, which can alter their distribution across different watershed zones [11]. Many studies have characterized TM pollution, toxicity, and enrichment by analyzing TM content in water bodies [12], sediments [13], and fish [14]. Additionally, source identification methods have been employed to trace the sources of TMs [15]. However, these approaches primarily focus on natural aquatic systems, neglecting the impacts of human-engineered structures like tidal gates. Tide gates, widely constructed for flood control and land management, profoundly alter sediment transport and TM dynamics in coastal watersheds. During the tidal gate closure, sediments from the catchment accumulate in the estuary, and this continued accumulation not only leads to peak concentrations of toxic metals [16] but also complicates the variable environmental conditions in the estuary [17], which undoubtedly adds more uncertainties and restrictions in identifying pollution risk factors and establishing prevention and control strategies at the watershed scale [18,19]. Therefore, the characteristics of distribution, the source, and the accumulation of TMs in sediments and their influencing factors need further research, especially in the tidal gate-controlled coastal watersheds.

Bacterial communities in sediment play a critical role in influencing the endogenous migration or enrichment of TMs [20]. These communities influence TM distribution and transformation through mechanisms such as assimilation [21], metabolism processes [22], and the alteration of environmental conditions [23,24]. Moreover, the precipitation process of TMs is substantially affected by fundamental chemical reactions associated with bacterial activity. For example, bacteria-mediated reduction reactions involving Fe compounds have been found to determine the distribution of TMs [25]. Conversely, TMs in sediments can also cause fluctuations in bacterial communities, not only for heavy metals [26] but even for light metals derived from parent material [27]. Thus, the dynamic relationship underscores the importance of bacterial communities in TM cycling and ecosystem health. Currently, bacterial communities are being increasingly employed as an effective tool for assessing and predicting ecosystem health [28], due to their high sensitivity to environmental changes [29] and adaptive mechanisms for ecosystem succession [30]. These studies are largely based on bacterial community composition or the alpha diversity of related functional genes to estimate the quality levels of ecosystems. For example, previous studies found that soil bacterial community composition is closely associated with land use types. Within the framework of a random forest model, it was possible to accurately classify land use and predict changes in soil quality parameters with an accuracy rate of 50 to 95% [26]. Furthermore, with increasing levels of environmental pollution, the functional gene community diversity detected by the comprehensive functional gene array (GeoChip5) significantly increased, and these changes can be directly used to predict nitrate pollution and ecosystem functions [31]. While bacterial communities have shown potential as bioindicators, their application in such anthropogenic-influenced ecosystems remains limited. This gap hinders the development of effective strategies for managing TM pollution in these complex environments. In our previous study, the functional metabolic abundance of bacterial communities was applied to the classification of space units in the coastal watershed [32]. Based on this, we hypothesize that due to the high sensitivity of bacterial communities to environmental changes [32], the functional metabolic abundance of bacterial communities could serve as a reliable ecological indicator for predicting TM pollution levels compared to traditional pollutant identification methods, offering a novel approach to ecosystem health assessment in tidal gate-controlled coastal watersheds.

Tianjin, the second-largest city in the Bohai Rim urban agglomeration of China, has numerous rivers, a substantial population, and developed industry and agriculture sectors. Over the past several decades, high-intensity land reclamation and the widespread implementation of tidal gates have significantly altered the natural dynamics of coastal ecosystems. These changes have led to varying levels of TM contamination in sediments. Early reports suggest that sediment accumulation and sewage discharge play a dominant role in TM contamination in the estuary [33]. However, the continued influence of tidal gates exacerbates TM deposition, resulting in persistent pollution risks; even the Chinese government’s integrated land use planning efforts have reduced direct land-based pollutant emissions [29]. Specifically, some TMs, such as Cd, As, Cr, Cu, and Zn, continue to pose potential risks in many estuarine and coastal sediments from Bohai Bay [34]. Therefore, there is an urgent need to investigate the migration mechanisms of TMs in this region, providing feasible approaches for similar tidal-gate-controlled coastal aquatic areas globally.

The Duliujian river watershed in Bohai Bay was selected as the study area due to its representative characteristics of tidal gate-controlled coastal systems. This region is a hotspot for intensive industrial and agricultural activities, making it vulnerable to TM contamination. The presence of distinct spatial units—the catchment area (CA), estuarine area (EA), and offshore area (OA)—provides a unique opportunity to investigate TM dynamics across different environmental gradients. This paper also discusses the interrelationships of the sediments of TM contents (Al, As, Cr, Cu, Fe, Mn, Ni, Sr, and Zn), the bacterial communities, and the major physicochemical properties. The objectives of our study are as follows: (1) to assess the spatial variation in TMs and bacterial communities in sediments in different spatial units at the watershed scale; (2) to explore the potential risk of enrichment, contamination, and accumulation of TMs in sediments, identify the main influencing factors, and analyze them retrospectively; and (3) to try to determine the contamination status of TMs by using the structure, diversity, and metabolism abundance of bacterial communities, and then, to discuss the feasibility, reliability, and practicability of bacterial communities as ecological indicators in identifying TM pollution and risk in the coastal watershed management.

## 2. Materials and Methods

### 2.1. Site Description

The Duliujian river watershed is an important flood channel in Tianjin, which belongs to the Daqing watershed system and is an artificial river channel that leads floods from the Daqing and Ziya rivers to the sea. It eventually flows into Bohai Bay in Northern China, which is the second largest bay, and accounts for one fifth of the total area of Bohai Bay. Due to the weak water exchange between Bohai Bay and the Yellow Sea, the physical self-cleaning capacity of Bohai Bay is very weak [35]. Many studies have shown that Bohai Bay has been subject to varying degrees of organic pollution [34,36]. To combat the continuing pollution of Bohai Bay, 12 major rivers that flow into Bohai Bay in Tianjin were set up with tidal gates in 2011.

### 2.2. Sampling Collection

**Figure 1:**
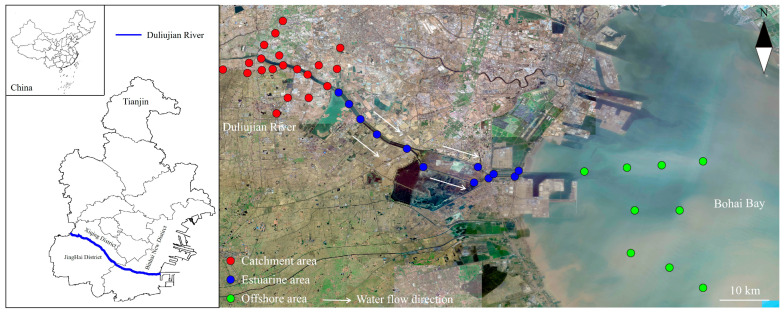
Sampling locations map in the catchment area (CA), estuarine area (EA), and offshore area (OA) of Duliujian river watershed in Bohai Bay (details in Table S1).

In this study, the sediment samples of the Duliujian river watershed were collected in September 2020. Based on the distribution of its mainstem and tributaries and the alignment of the coastal watershed, the Duliujian river watershed can be divided into three different spatial units, namely the CA, EA, and OA. The study area spans 120 km from the CA to the OA. At each sampling location, the surface sediment (0–20 cm) was collected using a mud sampler, and plant roots, shellfish, and other impurities were removed from each sediment sampling. Each sampling location was collected 3–5 times to form a comprehensive sample, thereby accurately estimating the actual situation of these sites. Some of the samples were placed in polyethylene plastic bags, transported back to the laboratory, and stored at −20 °C in a refrigerator for determination of physicochemical parameters and TM content, and the other samples were stored in dry ice buckets for the determination of 16s rRNA. A total of 41 sampling locations in the CA (20 locations), EA (12 locations), and OA (9 locations) were used to determine the physicochemical properties, and 35 sampling locations were used to determine the 16s rRNA in sediments. All sampling locations are shown in Figure 1.

### 2.3. Sample Processing, Sequencing, and Analysis

In this study, the physicochemical properties and TM content in sediments were determined by the following methods: air-dried samples were digested on a graphene electric heating plate for 2–3 h using a mixture of concentrated nitric acid and hydrofluoric acid, with concentrations of 1.42 g/mL (15 mL) and 1.16 g/mL (5 mL), respectively [37]. Then, the acid was removed and cooled to 2–3 mL, the cooling liquid was transferred into a 25 mL volumetric flask to ensure constant volume, and the TM contents and total P (TP) in the sediment samples were determined using inductively coupled plasma atomic emission spectrometry (VISTA-MPX, Varian, Palo, Alto. USA—2000) [38]. Furthermore, the air-dried samples were put into the flow analyzer to determine the content of total N (TN), the pH and salinity in sediment were determined by pH meter (Delta320, Mettler Toledo, Greifensee, Switzerland—2007) and conductivity meter (DDSJ-308A, LEICI, Shanghai, China—2010), the moisture content (MC) in the sediment was determined by the drying weighing method, and sediment organic matter (SOM) was determined by the potassium dichromate external heating method [32]. For 16s amplicon sequencing, its experimental process and analytical methods have been published [27], and have also been supplemented in the Supplementary Method S1.

### 2.4. Laboratory Analysis

#### 2.4.1. Enrichment Factor

The enrichment factor (EF) method was used to evaluate the TMs in this study [38].

(1) $$EF=\frac{\left(\frac{C_{M}}{C_{R}}\right)sample}{(\frac{C_{M}}{C_{R}})background}$$

where $\frac{C_{M}}{C_{R}}$ is the ratio of measured and reference metals of the sediment samples and their corresponding background values. The background values of TMs in Tianjin Province were used to assess pollution levels in this study area [39]. Specifically, the background values of Al, As, Cr, Cu, Fe, Mn, Ni, Sr, and Zn are 73,200, 9.6. 84.2, 28.8, 33,500, 33.3, 200, and 79.3 mg/kg, respectively.

If EF > 1, the metal is relatively enriched and influenced by human activities. If EF ≈ 1, it originates from the weathering of the crust. In addition to the use of EF values to evaluate the TM source, the enrichment degrees of the TMs were also classified according to the following EF values: EF < 2, slight enrichment; 2 ≤ EF < 5, moderate enrichment; 5 ≤ EF < 20, moderate and high enrichment; 20 ≤ EF < 40, high enrichment; and EF ≥ 40, severe enrichment [38].

#### 2.4.2. Geoaccumulation Index

The geoaccumulation index (I_geo_) was used to evaluate pollution [40].

(2) $$I_{geo}=\log_{2}\left[C_{n}/\left(k\times B_{n}\right)\right]$$

where *C_n_* is the measured content of metal in sediments, and *B_n_* is the geochemical background value of the TMs in sediments. The background values of the geological elements in Tianjin are described as TM background values, and *K* was 1.5. The pollution degree can be classified based on the following I_geo_ values: I_geo_ < 0, no pollution; 0 ≤ I_geo_ < 1, no pollution-medium pollution; 1 ≤ I_geo_ < 2, medium pollution; 2 ≤ I_geo_ < 3, medium pollution-heavy pollution; and 3 ≤ I_geo_ < 4, heavy pollution [40].

#### 2.4.3. Nemero Comprehensive Pollution Index

The Nemero comprehensive pollution index (PN) method was used to quantify the degree of TM pollution in this study [41].

(3) $$PN=\sqrt{\frac{{{\left(\frac{C_{i}}{S_{i}}\right)}^{2}}_{max}+{\left(\frac{1}{n}\sum_{i=1}^{n}\frac{C_{i}}{S_{i}}\right)}^{2}}{2}}$$

where PN is the synthesis evaluation score, *C_i_* is the measured content of the *i*-th element at a sampling location, and *S_i_* is the evaluation criterion of the *i*-th element. The evaluation criterion used in this study is based on the “China Environmental Quality Standard for Sediment Metals (HJ 1315—2023)” (pH > 7.5) [37]. Al, Fe, Mn, and Sr were evaluated based on the background values of territorial sediment elements (i.e., Al, Fe, Mn, and Sr are not specified in HJ 1315—2023). The *PN* value was graded into five categories: PN ≤ 0.7, safety; 0.7< PN ≤ 1.0, guard; 1.0 < PN ≤ 2.0, low pollution; 2.0 < PN ≤ 3.0, moderate pollution; and PN > 3.0, severe pollution.

### 2.5. Statistical Analysis

The “ggcor” R package was used to understand the correlation between the contents and related indices of TMs and environmental factors in sediments [42,43]. Furthermore, the “vegan” R package was used for non-metric multidimensional scaling (NMDS) and principal coordinate analysis (PCoA), which determined the difference in distribution characteristics in the bacterial communities and TM contents of different spatial units [44,45]. Finally, the “ggplot2” R package was used for visualization [46,47].

The relationship between TM–TM and TM–TM-related indices is presented using linear fitting. Principal component analysis (PCA) was used to analyze the sources of different TMs in sediments [41]. SourceTracker was used to analyze the source and sink relationships of TMs across different spatial units [48]. Furthermore, redundancy analysis (RDA) was used to determine the influencing factors of environmental factors, TM contents, and bacterial community parameters [49]. Variance partitioning analysis (VPA) was used to analyze the impact of TMs and bacterial dynamics on toxic metals and TM related indices [49].

In this study, data statistics and result visualization were completed using SPSS 25, Origin 2019, R 4.10, Canoco 5, and Surfer 15.

## 3. Results and Discussion

### 3.1. Physicochemical Properties in Sediments of Duliujian River Watershed

The trends of all physicochemical properties in sediments of the CA, EA, and OA were different (Table S2). ANOVA indicated that the pH and TP have the same trend significantly, which shows that EA > OA > CA (Table S2; *p* < 0.05). Moreover, the trend of MC is shown as OA > CA > EA, which has significant differences in different spatial units (Table S2; *p <* 0.05). In addition, TN (520.42 ± 56.16 mg/kg) and SOM (12,250.33 ± 432.28 mg/kg) in the EA are significantly lower than those in the CA and OA (Table S2; *p* < 0.05). Furthermore, salinity has a signifying gradient at the watershed scale, which shows that OA > EA > CA (Table S2; *p* < 0.05).

In terms of elemental cycling, N and P in the sediment show opposite trends. P is a sedimentary cycle [31], and more P accumulates in the EA probably due to the closure of the tidal gates. In contrast, N undergoes frequent ion exchange at the sediment water interface [49,50]. The closure of the tidal gates facilitates the release of N from the sediment into the overlying water. This process is likely to lead to the migration of N in the form of ions, consequently contributing to an elevated presence of nitrogen in offshore sediments [32]. Thus, the tidal gates in coastal rivers may have altered the habitat characteristics of the coastal watershed.

### 3.2. Distribution of TMs in Sediments of Duliujian River Watershed

**Figure 2:**
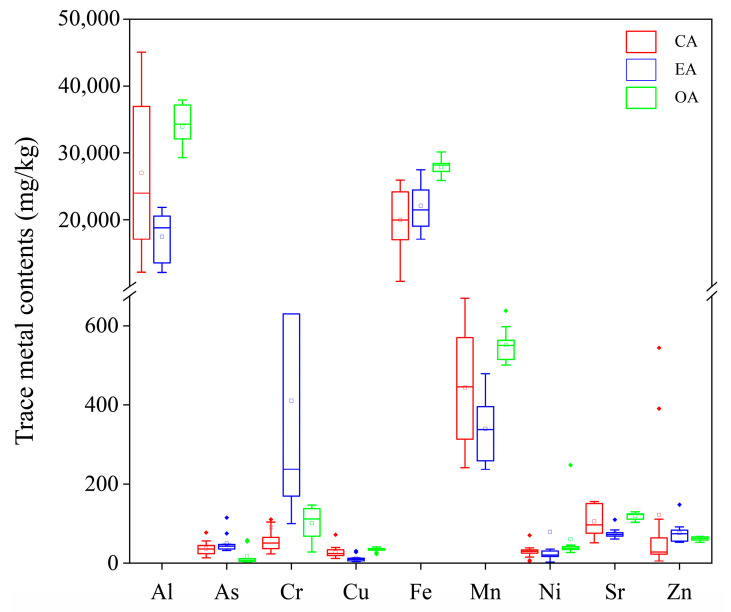
TM contents in the sediments of the CA, EA, and OA of Duliujian river watershed in Bohai Bay.

As expected, the content of TMs in the sediments varied considerably between the different spatial units affected by the tidal gates (Figure 2), as recorded by CA, EA, and OA as: 27,037.15, 17,489.76, and 33,943 mg/kg for Al; 37.30, 49.65, and 17.43 mg/kg for As; 90.81, 410.52, and 101.29 mg/kg for Cr; 27.64, 12.28, and 33.96 mg/kg for Cu; 20,006.09, 22,079.61, and 27,871.60 mg/kg for Fe; 443.75, 339.27, and 551.93 mg/kg for Mn; 29.39, 79.28, and 60.73 mg/kg for Ni; and 105.63, 75.27, and 119.42 mg/kg for Sr. In addition, PCoA showed that the TM content in the different spatial units did not show clear boundary characteristics (Figure S2), suggesting that the tidal gates may change the distribution and enrichment status of TMs in sediments and that the delineation of control units by TM content in sediments alone may become more complicated.

In the natural watershed, the TM distribution will show a clear gradient effect [51,52]. In this study, only Fe fulfilled the natural characteristics, with the remaining TMs showing enrichment at the EA or migration to the OA, indicating that the tidal gates at the EA may have altered the spatial distribution of TMs in sediments [53]. Moreover, there is a considerable distance from the CA to the OA. During the river’s long-distance transport, spatial differences arising from variations in sediment environmental parameters and human activities may be one of the factors influencing the changes in TM concentrations. This study further compares the results with those from other studies (Table S3). The results show that the concentrations of As and Cr have exceeded the average levels of rivers in China and globally. Moreover, the concentrations of As and Cr are clearly higher than those in the sediments of coastal rivers that are not influenced by tidal gates. Notably, this issue has been observed in other aquatic regions affected by tidal gates [54,55,56]. As toxic metals, high contents of As and Cr commonly occur in factories, farms, or livestock farms. Their levels are exacerbated by anthropogenic factors such as industrial pollution, effluent discharge, and human activities, with minimal occurrences in natural environments [57]. Furthermore, our previous studies have confirmed that the CA is the most anthropogenically disturbed ecological zone [32], but that high levels of locations in the sediment still occur in the EA. Therefore, we speculate that the As and Cr originating from the CA may have undergone migration to the EA through either water or sediment transport mechanisms. Owing to the strong retention effect of the tidal gates, it caused long periods of accumulation in the EA, which enhanced its sorption capacity, causing elevated levels in the sediment. However, the EA–OA is a complex interlacing zone of freshwater and saltwater where sediment ‘resuspension’ events, influenced by the water salt movement, may also be a major cause of the uneven distribution of TMs in sediments [58]. Therefore, the source of TMs needs to be further determined.

### 3.3. Pollution and Enrichment Indices of TMs in Sediments

**Figure 3:**
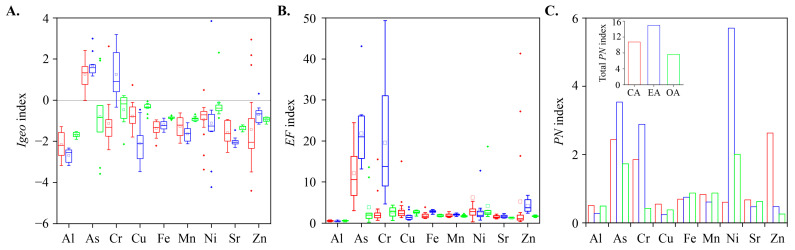
(**A**) Geoaccumulation index (I_geo_), (**B**) enrichment factors (EF), and (**C**) Nemerow pollution index of TMs in the sediments of the CA, EA, and OA of the Duliujian river watershed in Bohai Bay.

Several studies have shown that the I_geo_ is a reliable parameter to reflect the accumulation degree and pollution status of TMs in sediments based on a pollution intensity classification [59,60]. The I_geo_ values are compared in Figure 3A. Except for the values of As and Cr in the EA, and Ni in the EA and OA, the values of the other TMs in the three areas are less than 0, which can be characterized as a pollution-free state. The I_geo_ of As is grade 2 (medium pollution) in the CA and EA and grade 1 (non-medium pollution) in the OA. The I_geo_ of Cr in the EA is grade 2 (moderate pollution). The I_geo_ of Ni is grade 1 (medium pollution) in the EA and OA. The findings indicate that the I_geo_ for different metals have significant differences. In particular, the impact on As and Cr enrichment remains notably high in the EA. ANOVA shows significant differences in As, Cr, Al, Fe, and Mn in the three regions (*p* < 0.05). The I_geo_ of As and Cr in the EA are significantly higher than those in the other two regions (*p* < 0.05), suggesting a certain degree of accumulation of As and Cr and potential ecological risks. In addition, the I_geo_ of Al, Fe, and Mn in the OA are significantly higher than those in the other two regions (*p* < 0.05), which may be related to the varied sources of the TMs.

Except for the patterns for Al, Cu, and Zn, the EF of other TMs in the EA are higher than those in the other two regions (Figure 3B). According to the classification of EF values, As is highly enriched in the CA and EA, and moderately enriched in the OA. The EF value was easily affected by human factors, such as the petroleum industry and river regulation, which may lead to the accumulation of As in sediments of different watersheds [61]. The EF of Cr in the EA belongs to the medium and high enrichment levels and it belongs to the medium enrichment level in the CA and OA. The EF values of Cr, Cu, Ni, and Zn are higher than in the CA, which belongs to moderate enrichment. Among them, As and Cr are potential toxic metal pollution sources. When the exogenous input or bioaccumulation release enters the overlying water, the precipitation process will occur. Furthermore, when the physical and chemical properties in the sediment change, the accumulated TMs will also be released into the overlying water, making the sediment an endogenous pollution source and causing a large degree of endogenous pollution [62]. Therefore, great attention should be paid to the As and Cr pollution in the whole study area.

The PN was used to evaluate the TM pollution degree in the study area and Figure 3C illustrates the PN of each TM in the three different regions. The PN of Al, Cu, Fe, Mn, and Sr in three regions are all lower than 1, indicating that no pollution was found for these TMs. The PN of As, Cr, and Ni in the EA all exceed 2, indicating severe pollution. This is suggestive of potential influences arising from human activities, atmospheric factors, acid deposition, and bioaccumulation in the vicinity of the EA. Consequently, there is a pressing need for targeted monitoring to identify and address specific sources of sediment pollution in the EA. The PN of Zn in the CA is 2.6, indicating moderate pollution, which may be attributed to wetland reclamation and agricultural activity control (i.e., fertilization and sewage irrigation). Furthermore, the PN of Fe and Mn is approximately 0.7, indicating a pollution-free state. A possible explanation is that Fe and Mn mainly originate from parent materials. In addition, the total PN values at the watershed show EA (14.93) > CA (10.75) > OA (7.64), suggesting that the tidal gates would significantly mitigate the potential risk of TMs in sediments in the OA, but have the potential to exacerbate TMs pollution in the EA.

### 3.4. Source Analysis for TMs and Their Abiotic Influencing Factors

**Figure 4:**
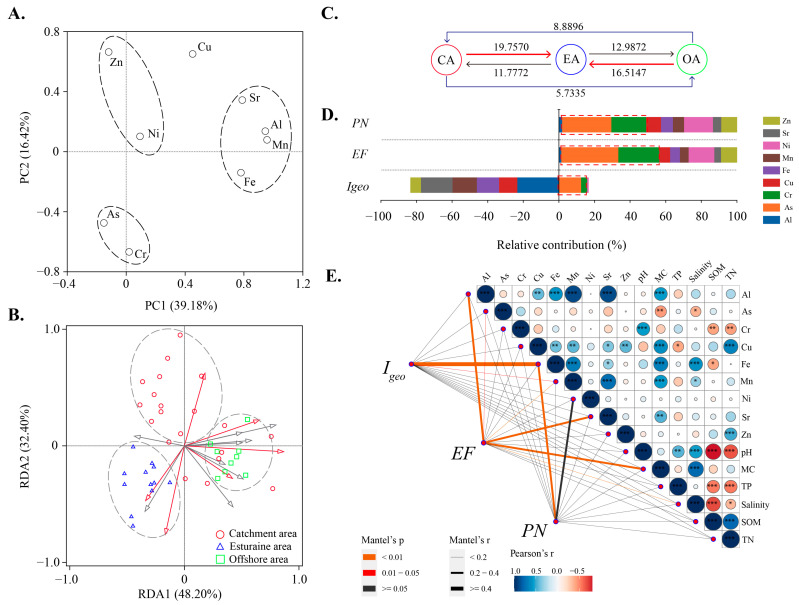
(**A**). Principal component analysis of TMs in the different spatial units. (**B**) RDA of TM with environmental factors. (**C**) SourceTracker analysis of the sink and source relationships of TMs in different spatial units. The values represent the total exchange potential. (**D**) The degree of contribution of TMs in sediments to each index. The red box represents the degree of contributions of As and Cr. (**E**) Paired comparison of environmental factors and TMs with the TM related indices. The color gradient and circle size represent the Spearman correlation coefficients; the width of the line represents the degree of correlations among potential risks, TMs, and environmental factors. Asterisks denote statistically significant differences (***, *p* < 0.001; **, *p* < 0.01; *, *p* < 0.05).

The accumulation risk level of TMs is not only related to their concentrations but also their sources [63]. PCA is usually used to identify the pollutant source [64,65,66]. The results showed that the first two principal components can explain 55.61% of the variable changes (Figure 4A; Table S4). The first principal component (PC1) represents the natural factors [67], and is mainly loaded by Mn (0.96), Al (0.95), Sr (0.79), and Fe (0.78) in this study, explaining 39.2% of the common variance (Figure 4A; Table S4). Furthermore, Al, Fe, Mn, and Sr show a significant positive correlation (Figure 4E; *p* < 0.001), indicating a similar source of these TMs. The presence of Al, Fe, Sr, and Mn suggests an association of these elements with rock weathering, lithology, or particle size characteristics [68,69,70]. PC1 appears to delineate the gradient between continental and oceanic realms or particle-size distribution [41]. Therefore, it can be inferred that Al, Fe, and Mn likely originated primarily from parent materials. However, due to wastewater discharge and industrial production, the influence of human activities may contribute to the elevated correlation between these TMs in certain regions. Future studies need to incorporate additional data of anthropogenic influences to clarify this aspect.

The second principal component (PC2) represents human factors [67] and is negatively correlated with As and Cr but positively correlated with Zn and Ni (Figure 4A; Table S4) in this study. The concentrations of these four TMs are predominantly shaped by anthropogenic disturbance, with minimal natural occurrences. The findings highlight the association of these TMs with human interference. However, it is noteworthy that the origins of the paired trace metals (As and Cr; Zn and Ni) exhibit divergence, indicating that these metal pairs have a different source. First, Zn and TN have a significant positive correlation (Figure 4B; *p <* 0.001), indicating that Zn may have originated from wetland reclamation or agricultural activity control (i.e., fertilization and sewage irrigation), while Ni and Zn have a similar origin. Therefore, coastal wetland management should perhaps be strengthened. Second, for As and Cr, our above speculations were verified, and the source of As and Cr may be due to anthropogenic activities at the CA that increase the entry of these two TMs into the overlying water. In addition, As and Cr are the TMs that account for the largest proportion of sediment in the various indices calculated for TM content (Figure 4D). Combined with SourceTracker analysis (Figure 4C), when the CA and OA serve as TM sources and the EA acts as a sink, the potential for the spatial transport of TMs from source to sink is clearly greater than that were EA to be the source. Furthermore, TMs show the least migration potential from the CA to OA or from the OA to CA (Figure 4C). These results suggest that the presence of tidal gates may alter the spatial distribution of TMs, particularly, As and Cr. There are two possible explanations for this phenomenon. First, the operation of tidal gates affects the redox environment of the water. Closing the gates may cause water retention, creating a higher redox environment, which can lead to the increase in TM concentrations in the sediments. For example, studies have shown that the cycling of As at the groundwater surface water interface in tidal channels is controlled by hydrological conditions, with redox conditions varying across tidal and seasonal timescales, affecting the mobility of As [71]. Second, tidal gates control the mixing of freshwater and seawater, altering salinity gradients. Changes in salinity affect the solubility and adsorption behavior of TMs, thereby influencing their distribution in the sediments. Research has found that TMs exhibit a clear response pattern at the tidal and salinity interfaces in estuarine areas, with the salinity effect significantly impacting the partition coefficients of metals such as As and Cr [72].

Incorporating environmental factors and TM into the RDA framework, it can be seen that different aquatic regions have distinct boundary features between them, suggesting that different environmental factors may be the main reasons influencing the distribution of TMs (Figure 4B; Table S5). Next, the correlation heatmap revealed the relationship between the contents of TMs and the physicochemical properties in sediments at the coastal watershed (Figure 4E). TMs will be affected by various physical and chemical properties [73], with varying degrees of environmental factors affecting their transport rates. For As, there was a significant negative correlation with salinity (Figure 4E; *p* < 0.05). In this study, there was a gradual increase in salinity from the CA to the OA, and its increase may have inhibited the migration ability of As. Salinity is the most important factor and an increase in salinity usually means a decrease in the rate of migration and sedimentation of metal ions, as it can alter the free metal ion activity and, thus, the bioavailability and toxicity of TMs in coastal systems [74]. For example, previous studies have found that as salinity increased, the uptake of TMs by brackish water organisms became less efficient, resulting in TMs being less likely to be deposited in coastal systems [75]. Some scholars have specified TM ions (Cd^2+^) and saline organisms (*Bahia pigmentia*) to study the effect of salinity on uptake efficiency, and morphological modeling revealed a 25% reduction in the percentage of free ions [76]. Furthermore, for Cr, there was a positive correlation with pH (Figure 4E; *p* < 0.05), with elevated pH causing the accumulation of TMs in the sediment [77]. Therefore, the influence given by the environment of the estuary may be the main cause of potential As and Cr pollution in the sediments.

### 3.5. Correlation Analysis Between Potential Risks and Environmental Factors

**Figure 5:**
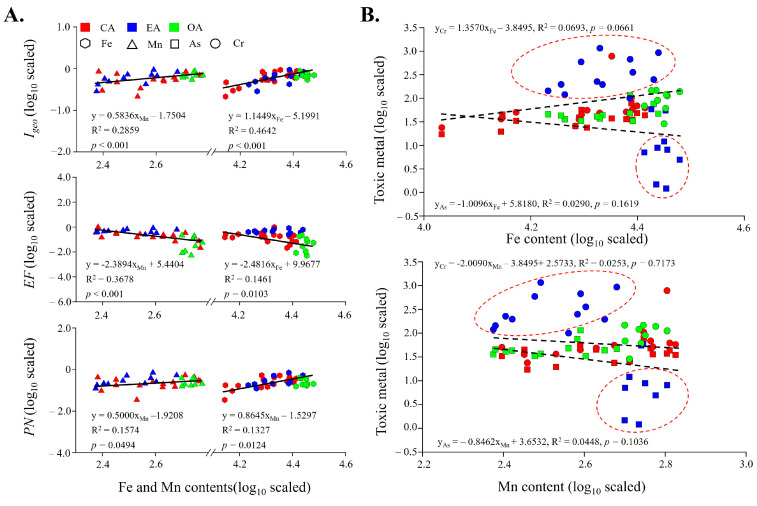
(**A**) Regression analysis for pairwise combination of Fe and Mn with TM correlation indices, and (**B**) pairwise combination of Fe and Mn with toxic metals (As and Cr). Axes are log_10_ scaled.

The correlation coefficients, such as I_geo_, EF, and the PN of TMs, were used as variables to test their correlations with environmental factors and individual TMs. Al, Fe, and Mn were found to have significant correlations with those indices. In this study, Al was used as the reference TM for geochemical normalization because its geochemistry was like that of many TMs and its natural content tended to be stable [78]. Then, taking Al as a reference coefficient to calculate the *EF* in sediments might be the main reason for the significant correlation between EF and Al. Furthermore, iron-manganese oxides are the main forms of Fe and Mn in sediments and can improve the effectiveness of metal pollutants under certain redox changes in nature [79]. It can even be considered a key metal that indicates the pollution and enrichment status of TM in sediment [13]. In this study, Fe and Mn are generally derived from parent material (Figure 4A), the main constituents of which are primary minerals (such as quartz, feldspar, and mica), and their formation is largely dependent on the composition of the sediment and other TMs in the environment [80]. Furthermore, this study attempts to use Fe and Mn as monitoring factors for the pollution and enrichment of TM, and to linearly fit the correlation indices of TM. The results indicate that Fe and Mn have good relationships with different indices of TM (Figure 5A), indicating that the content of Fe and Mn in sediment may monitor sediment metals status to some extent. Next, this study made an in-depth attempt to investigate the ability of Fe and Mn as monitoring factors. In theory, they can characterize the occurrence states of As and Cr in sediments. The oxides of the Fe and the Mn had strong oxidation and adsorption capacities for As and Cr. Under oxidation conditions, they can effectively oxidize dissolved Cr^3+^ and As^3+^, while most of the newly formed Cr^6+^ and As^5+^ will be adsorbed on the metal surface and fixed [81,82,83]. Furthermore, EA was affected by the tide, and some sediments would be exposed to the air for a long time, resulting in the adsorption of exogenous As and Cr on the surface of Fe and Mn aboveground oxide. However, the correlation between pairs of TMs did not reach the desired results (Figure 5B). It is easy to see that some locations in EA have strayed, resulting in a shift in the fitting results, suggesting that the construction of tidal gates in EA has altered the natural characteristics of toxic metals in the coastal watershed, which may complicate the monitoring efforts. In addition, As and Cr, as high-concentration toxic metals, pose potential ecological and human health risks due to the unique environmental characteristics at EA. The increase in As and Cr content in the EA indicates that the presence of tidal gates has severely affected the spatial distribution characteristics of toxic metals. As is a known carcinogen and exhibits the highest toxicity in its inorganic As. Under the reducing conditions, it can be desorbed from sediment particles, thereby increasing its bioavailability and toxicity in aquatic systems. Research has shown that an increase in As content in sediments can cause toxic effects on aquatic organisms through food chain bioaccumulation, while also increasing the risk of human exposure through drinking water [84]. Moreover, Cr exhibits high toxicity and mobility in an oxidizing environment. Its accumulation through the food chain in aquatic systems may pose a severe risk to the growth of aquatic organisms [85]. Therefore, there is an urgent need for innovative methods to identify TM pollution affected by tidal gates in coastal watersheds.

### 3.6. Bacterial Communities and Their Influencing Factors at the Coastal Watershed

**Figure 6:**
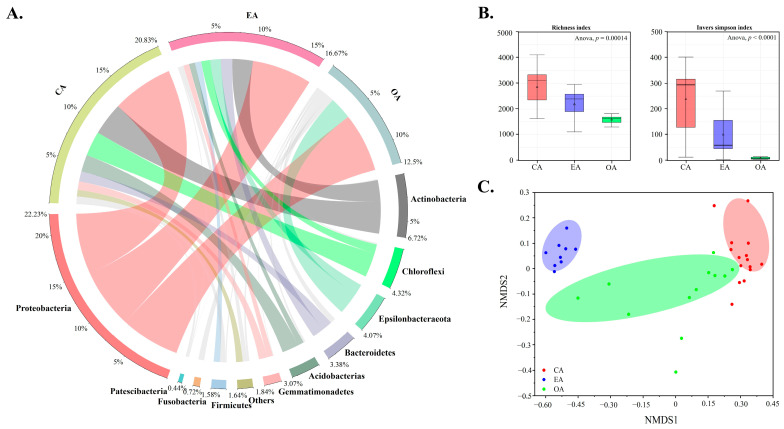
Changes in community composition (**A**) and diversity (**B**) of bacterial communities and NMDS analysis (**C**) for determining differences in bacterial communities at the watershed scale.

As the four most dominant phyla at the watershed scale, the relative abundance of *Proteobacteria*, *Actinobacteria*, *Chloroflexi*, and *Epsilombacteraeot* collectively represent over 70% of the total relative abundance (Figure 6A and Table S6). Moreover, the bacterial community’s alpha diversity and functional abundance exhibited distinct characteristics across the basin, gradually decreasing from the CA to the OA (Figure 6B and Figure S2; *p* < 0.05).

Additionally, the NMDS results revealed clear boundary distinctions among bacterial communities across the coastal watershed, indicating variations in their composition across different spatial units (Figure 6C). Furthermore, RDA was used to determine the influence of different variables in the sediment of the different spatial units on the structure, diversity, and metabolism abundance of the bacterial community (Figure S3); it showed that salinity (57.7% and 64.2%), MC (53.9 and 48.2%), SOM (27.3 and 33.1%), and pH (18.9 and 21.0%) were found to be most influential (Tables S7–S9).

There is considerable variation in the pattern of bacterial communities in different spatial units, and the diversity and metabolism abundance of bacterial communities have clear watershed characteristics. In general, bacterial community structure is highly sensitive, with changes in the local environment shaping the structure of similar bacterial communities [32]. In this study, the EA was the most heavily contaminated area by the accumulation and pollution of TMs, such as As and Cr. However, the diversity and metabolism abundance of the bacterial community were much higher in the CA than in the EA. Could this be due to the higher contamination level in the CA compared to the EA? There are two possible reasons for this phenomenon. First, the changing characteristics of the overlying water environment need to be considered; previous studies have confirmed that CA-overlying waters are more eutrophic than EA waters [32] and that high eutrophic levels drive the enhanced functionality and primary productivity of bacterial communities, possibly creating new bacterial ecological niches [86]. Second, salinity is a major driver in structuring bacterial communities. Previous studies have compared changes in microbial diversity from freshwater to marine salinity trajectories and found that microbial diversity was progressively lower moving from the freshwater end of the river to the coast [87]. It is worth noting that the EA is a complex dynamic area subject to tidal action resulting in the alternation of fresh and salty water. Although the variation in bacterial communities in coastal river sediments is manipulated by salinity, it is not systematic, with a greater abundance of genes encoding heterotrophic processes compared to the river and marine genes, and certain groups of bacteria may exhibit greater abundance profiles [88].

### 3.7. The Indicative Role of Bacterial Community on TMs in Duliujian River Watershed

**Figure 7:**
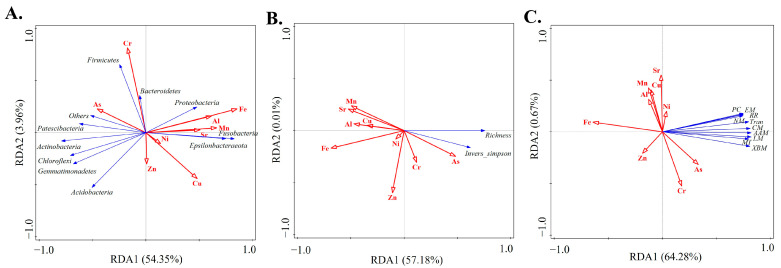
RDA of TM with bacterial dynamics. (**A**) TM with dominant abundance of bacterial community; (**B**) TM with bacterial diversity; (**C**) TM with bacterial metabolism abundance. Among them, PC represents the poorly characterized; EM represents the energy metabolism; RR represents the replication and repair; NM represents the nucleotide metabolism; Tran represents the translation; CM represents the carbohydrate metabolism; AAM represents the amino acid metabolism; LM represents the lipid metabolism; MT represents the membrane transport; XBM represents the xenobiotics biodegradation and metabolism.

**Figure 8:**
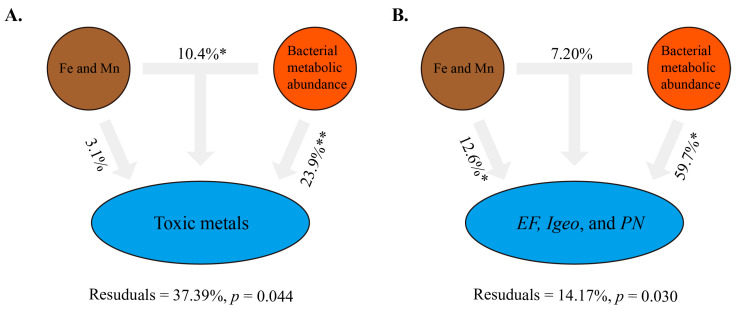
VPA of traditional TM identification (Fe and Mn) and bacterial metabolic abundance for toxic metals (**A**) and TM-related indices (**B**). Among them, * and ** represent *p* < 0.05 and *p* < 0.01, respectively. Toxic metals contain As and Cr.

Within the framework of RDA, the response relationship between bacterial community structure, diversity, and metabolism abundance in different spatial units was investigated by using nine trace metals as influencing factors. The results showed that RDA1 and RDA2 explained 58.31, 57.19, and 64.95% (Figure 7A–C) of bacterial community structure, diversity, and metabolic abundance. Notably, Fe and Mn were the key TMs influencing changes in structure, diversity, and metabolism abundance as analyzed by Monte Carlo (Tables S10–S12), suggesting that the dynamics of bacterial communities at the coastal watershed can be influenced by the mediating effects of Fe and Mn from the parent material. It is worth noting that only the metabolic abundance of bacterial communities is correlated with As and Cr. Furthermore, this study further compares the accuracy of bacterial metabolic abundance and traditional pollutant identification methods in the framework of VPA (Figure 8). The results showed that bacterial metabolic abundance could explain 23.9 (Figure 8A; *p* < 0.01) and 59.7% (Figure 8B; *p* < 0.05) of the changes in toxic metals and TM related indices, respectively. However, Fe and Mn can only significantly explain the changes in TM related indices (*p* < 0.05), but they do not seem to fully explain the changing trends of toxic metals (*p* > 0.05). These results indicate that dynamic changes in the metabolic abundance of bacterial communities as a monitoring factor for assessing the state of contamination, accumulation, and enrichment of TM in sediments are a better approach in tidal gate-influenced coastal river.

Introducing bioindicators into future river monitoring processes to better estimate TM pollution in aquatic regions is feasible, because of two factors. First, the construction of gates may lead to instability in water levels and tidal variations, which may affect the distribution and transport of TMs in the sediments [89,90]. However, the synchronized adaptations exhibited by bacterial communities allow them to quickly adapt and respond to ecosystem health [91]. Bacteria inevitably encounter various elements in the natural environment. Mn^4+^, Fe^2+^, and Fe^3+^ can act as biological donors or acceptors and can participate at the same time. With the participation of bacterial communities, Fe^2+^ and Mn^2+^ will be oxidized into Fe^3+^ and Mn^3+^, and other bound colloids are deposited in the substrate, increasing the content of Fe and Mn in the substrate. Biological reduction has always been regarded as an important metabolic process. It controls the migration and transformation of TMs in sediments. At the same time, in the natural environment, TMs are sufficient to meet the physiological needs of bacteria at lower concentrations [58], and when TMs accumulate and contaminate, they may undergo the same changes as sediment contamination to better adapt to the environment. Second, given the heightened sensitivity of bacterial communities to environmental changes, their metabolic activity is inevitably impacted when their habitat faces stress from specific pollutants. In cases where these pollutants are decomposable by community members, the metabolic abundance increases to meet the heightened energy demands required for pollutant breakdown. However, if the concentration of pollutants exceeds the decomposition capabilities of the community, some bacteria may diminish in number or vanish entirely, leading to the loss of specific functional metabolic pathways. Despite this, members with greater adaptability may counter environmental disturbances through mechanisms like dormancy, evolving new metabolic pathways to restore their original activity levels. These functional pathway changes can still be rapidly detected. Therefore, during regional pollution assessments, especially in the preliminary background investigation and final evaluation phases, we strongly advocate for the use of bacterial metabolic abundance as a critical biological indicator. By analyzing the metabolic dynamics of bacterial communities, not only can the effectiveness of project implementations be assessed, but targeted management strategies can also be developed based on the accumulation of pollutants. Nevertheless, we must acknowledge that the conclusion of this study may have certain limitations. In particular, the concentration of TMs is significantly influenced by seasonal variations in aquatic areas. Previous research has shown that TM concentrations are typically lower during the dry season compared to the rainy season, both in overlying water and sediments [92]. Therefore, this study may have time constraints, which could bias the findings. To address these limitations, future studies should conduct long-term monitoring across different seasons and establish additional sampling sites along coastal rivers regulated by tidal gates for experimental analysis. This approach will facilitate a more comprehensive understanding of TM behavior and distribution, thereby enhancing the scientific rigor and applicability of the results.

## 4. Conclusions

This study highlighted the intricate dynamics between TMs and bacterial communities in tidal gate-affected coastal watersheds. It revealed that the EA exhibited the highest TM pollution risk, particularly with As and Cr, a situation exacerbated by the blocking effects of tidal gates, which promotes sediment accumulation. This issue may also reflect challenges faced by many tidal gate-controlled coastal rivers globally. In such complex environments, traditional pollutant identification methods may struggle to accurately monitor toxic metals. Fortunately, this study identified that the functional metabolic abundance of bacterial communities serves as a reliable indicator of TM contamination levels, making it a key ecological indicator for ecosystem health assessment and pollution management in future coastal watershed management. This underscores the feasibility and reliability of incorporating bacterial community dynamics into environmental management practices to more effectively track and monitor TM pollution, especially where traditional chemical monitoring proves insufficient. In addition, we recommend that future studies focus on the resilience and functional adaptability of bacterial communities under different TM loads and environmental conditions, which could provide new insights into ecosystem recovery and management strategies. Overall, this finding provides a foundational understanding of TM–bacteria interactions, paving the way for more targeted and efficient environmental management practices in coastal ecosystems.

## Data Availability

The data that support the findings of this study are available from the corresponding author upon reasonable request.

## Supplementary Materials

The following supporting information can be downloaded at: https://www.mdpi.com/article/10.3390/toxics12120839/s1, Method S1. Details in 16S rRNA sequencing and analysis. Table S1. The details of latitude and longitude information from different groups in the study area [29,93,94]. Table S2. The physicochemical properties of sediments in the CA, EA, and OA of Duliujian coastal watershed in Bohai Bay. Table S3. Comparison of TM concentration in sediments between this study and other studies (mg/kg) [54,55,56,95,96,97,98]. Table S4. Rotated component matrix and initial eigenvalues of principal component analysis of TMs in the CA, EA, and OA of Duliujian coastal watershed in Bohai Bay. Table S5. Important ranking for physicochemical properties and TM in different space units at the watershed scale. Table S6. Average relative abundance of dominant bacteria phylum in sediment samples. Table S7. Importance ranking of environmental factors in sediment to explain the overall characteristics of dominant phylum abundance of bacterial communities in different watershed spatial units. Table S8. Importance ranking of environmental factors in sediment to explain the overall characteristics of bacterial diversity in different watershed spatial units. Table S9. Importance ranking of environmental factors in sediment to explain the overall characteristics of bacterial metabolism abundance in different watershed spatial units. Table S10. Importance ranking of trace metals in sediment to explain the change in the dominant phylum abundance of bacterial community in different spatial units at the watershed scale. Table S11. Importance ranking of trace metals in sediment to explain the change in the diversity of bacterial community in different spatial units at the watershed scale. Table S12. Importance ranking of trace metals in sediment to explain the change in the metabolism abundance of bacterial community in different spatial units at the watershed scale. Figure S1. PCoA of the variability between TMs contents in sediments from different spatial units at the watershed scale. Figure S2. Metabolic abundance of bacterial communities on KEGG database in different spatial. Among them, one-way ANOVA was used to test for variability in metabolic abundance at the watershed scale. Figure S3. RDA of environmental factors with bacterial community dynamics. A. Effect of environmental factors on the dominant community abundance; B. effect of environmental factors on the diversity; C. effect of environmental factors on the metabolism abundance.
