# Supplementary material for: Using Sediment Bacterial Communities to Predict Trace Metal Pollution Risk in Coastal Environment Management: Feasibility, Reliability, and Practicability
Source: Toxics. 2024 Nov 22;12(12):839. doi: 10.3390/toxics12120839 (PMC11679552; doi:10.3390/toxics12120839)
Supplement: Supplementary file 1 [file toxics-12-00839-s001.zip › toxics-3299193-supplementary.pdf]

Supplementary materials

Method S1

16S rRNA sequencing and analysis. In this study, an Illumina platform with 16S rRNA technology was used to analysis the bacterial community characteristics for sediment samples. First, the 1% ager gel was used to electrophoretic detection to complete the extraction of genomic DNA. Second, the amplification region is V3-V4, and specific primers with borcode are used for splicing. Among them, the forward and reverse primers are 341F (CCTAYGGGRBGCASCAG) and 806R (GGACTACNNGGGTATCTAAT). According to the concentration of PCR products, equal concentration samples were mixed and purified using 2% agarose gel electrophoresis after thorough mixing, the GeneJET gel recovery kit (USA, Thermo Scientific) was used for product purification. Moreover, TruSeq® DNA PCR-free sample preparation kit was used fir library construction. After passing Qubit quantification and library detection, the constructed library was sequenced using Illumina Novaseq6000. For the above steps, they were all completed by applied protein technology (Shanghai, China).

For analysis processes of bacterial community, the greengenes database (http://greengenes.secondgenome.com/) was used for species annotation of bacterial communities to summary the total OTU abundance table with a consistency of 97% [93]. Furthermore, the "vegan" R package was used to calculate the alpha diversity of bacterial community [29], and the developed PICRUSt in imageGP (http://www.ehibio.com/ImageGP/) was used to predict the functional metabolism of bacterial communities [94].

Table. S1. The details of latitude and longitude information from different groups in the study area.

Collected regions	Name	longitude (°)	latitude (°)
CA	1	116.9820	39.1326
	2	116.9655	39.1096
	3	116.9391	39.0797
	4	116.9746	39.0774
	5	117.0623	39.0247
	6	117.0695	39.0624
	7	117.1233	39.0644
	8	116.9158	39.0581
	9	116.9499	39.0395
	10	116.9898	39.0265
	11	117.0268	39.0115
	12	117.0563	38.9973
	13	116.9113	39.0377
	14	116.8713	39.0185
	15	116.8922	38.9830
	16	116.9493	39.0157
	17	116.9271	38.9895
	18	117.0030	38.9666
	19	117.0481	38.9570
	20	117.0387	38.8367
EA	21	117.0735	38.9850
	22	117.1173	38.9635
	23	117.1581	38.9189
	24	117.1953	38.8773
	25	117.2725	38.8328
	26	117.3475	38.7763
	27	117.4544	38.7882
	28	117.4492	38.7446
	29	117.4818	38.7708
	30	117.4833	38.7588
	31	117.5582	38.7709
	32	117.5591	38.7622

Collected regions	Name	longitude (°)	latitude (°)
	33	117.6481	38.7867
	34	117.7522	38.8061
	35	117.8458	38.8264
	36	117.9634	38.8518
OA	37	117.7642	38.7642
	38	117.8900	38.7567
	39	117.7739	38.7148
	40	117.8645	38.6781
	41	117.9851	38.6399

Notes: CA represents the catchment area. EA represents the estuarine area. OA represents the offshore area.

Table S2. The physicochemical properties of sediments in the CA, EA, and OA of Duliujian coastal watershed in Bohai Bay.

Regions	pH	Salinity(%)	SOM(mg/kg)	TP (mg/kg)	`TN (mg/kg)	MC (%)
CA	7.02±0.04^{b}	0.03±0.01^{c}	31295.68±363.89^{a}	428.52±51.02^{b}	1317.25±121.93^{a}	29.71±1.22^{b}
EA	7.41±0.05^{a}	0.11±0.02^{b}	12250.33±432.28^{b}	705.59±28.11^{a}	520.42±56.16^{b}	24.44±0.95^{c}
OA	7.39±0.01^{a}	0.39±0.01^{a}	9698.00±354.65^{b}	514.29±5.62^{b}	813.33±29.04^{b}	49.36±1.19^{a}

Notes: a, b, and c represent the significant relationship of different spatial units. The values mean the average ± SE.

Table. S3. Comparison of TM concentration in sediments between this study and other studies (mg/kg).

Regions		Al	As	Cr	Cu	Fe	Mn	Ni	Sr	Zn	Reference
This study	Duliujian river watershed	25758.77	36.55	186.69	24.53	22339.55	436.92	50.87	99.77	95.63	
Coastal regions	Samoa coastal areas	31000	3.6	-	39.0	-	-	161	-	98.5	[95]
	South Yellow Sea	-	11.68	67.33	21.98	-	-	34.87	-	73.03	[96]
	Weihai coastal area	-	9	60.1	11.6	-	-	-	-	40	[97]
Tide-controlled coastal regions	Hackensack River	-	-	207.52	136.17	-	548.09	50.94	-	328.59	[54]
	Jinshan river	-	30.49	245.30	24.42	-	-	-	-	143.12	[55]
	Laizhou Bay	-	35.87	30.51	11.68	-	-	19.89	-	40.49	[56]
Other regions	River in China	-	14.39	76.58	90.98	-	-	-	-	194.09	[98]
	Rivers in world	-	9.06	51.51	191.11	-	-	-	-	388.69	

Notes: "-" represents that the selected research did not determine this TM.

Table S4. Rotated component matrix and initial eigenvalues of principal component analysis of TMs in the CA, EA, and OA of Duliujian coastal watershed in Bohai Bay.

Heavy metals	Rotating principal component		Component	Initial eigenvalue
	First principal component (PC1)	Second principal component (PC2)		Total/%
Al	0.946	0.136	1	39.18
As	-0.151	-0.476	2	55.61
Cr	0.021	-0.668	3	68.69
Cu	0.453	0.651	4	79.93
Fe	0.780	-0.140	5	88.10
Mn	0.958	0.078	6	95.4
Ni	0.095	0.101	7	98.51
Sr	0.789	0.343	8	99.67
Zn	-0.118	0.664	9	100

Table. S5. Important ranking for physicochemical properties and TM in different space units at the watershed scale.

Environmental factors	Importance	Explanation (%)	F	*P*
MC	1	17.60	8.354	**0.002**
TN	2	13.30	5.991	**0.002**
pH	3	10.90	4.761	**0.002**
SOM	4	9.80	4.252	**0.004**
Salinity	5	8.60	3.674	**0.006**
TP	6	5.80	2.384	**0.034**

Table S6. Average relative abundance of dominant bacteria phylum in sediment samples.

	Proteobacteria	*Actinobacteria*	*Chloroflexi*	*Epsilonbacteraeota*	*Bacteroidetes*	*Acidobacteria*	*Gemmatimonadetes*	*Firmicutes*	*Fusobacteria*	*Patescibacteria*	*Others*
CA	36.333±2.082	19.505±1.998	14.626±1.676	0.077±0.021	6.101±0.667	9.995±0.993	5.001±0.852	3.076±0.934	0.013±0.003	1.361±0.245	3.912±0.432
EA	44.137±3.503	15.26±2.589	6.630±0.989	7.767±5.949	8.578±1.198	4.401±1.095	4.317±0.817	4.611±1.589	0.022±0.004	0.807±0.221	3.470±0.527
OA	58.415±4.847	0.906±0.285	1.365±0.093	22.061±5.439	5.401±0.651	2.018±0.180	0.640±0.076	1.328±0.217	5.704±0.096	0.159±0.022	2.002±0.095
P	**0.001**	**0.001**	**0.001**	**0.004**	**0.049**	**0.001**	**0.002**	0.187	**0.001**	**0.003**	**0.018**

Significant results are shown in bold.

Table. S7. Importance ranking of environmental factors in sediment to explain the overall characteristics of dominant phylum abundance of bacterial communities in different watershed spatial units.

Environment factors	Important ranking	Explanation (%)	F	*P*
Salinity	1	57.7	46.3	**0.002**
MC	2	53.9	39.8	**0.002**
SOM	3	27.3	12.8	**0.002**
pH	4	18.9	7.9	**0.004**
TN	5	4.3	1.5	0.252
TP	6	0.8	0.3	0.808

Significant results are shown in bold.

Table. S8. Importance ranking of environmental factors in sediment to explain the overall characteristics of bacterial diversity in different watershed spatial units.

Environment factors	Important ranking	Explanation (%)	F	*P*
Salinity	1	64.2	60.9	**0.002**
MC	2	48.2	31.6	**0.002**
SOM	3	33.1	16.8	**0.004**
pH	4	21.0	9	**0.008**
TN	5	6.4	2.3	0.16
TP	6	1.0	0.3	0.536

Significant results are shown in bold.

Table. S9. Importance ranking of environmental factors in sediment to explain the overall characteristics of bacterial metabolism abundance in different watershed spatial units.

Environment factors	Important ranking	Explanation (%)	F	*P*
Salinity	1	37.8	20.6	**0.002**
SOM	2	30.1	14.7	**0.002**
MC	3	21.1	9.1	**0.006**
pH	4	20.6	8.8	**0.008**
TN	5	7.9	2.9	0.104
TP	6	7.1	2.6	0.106

Significant results are shown in bold.

Table. S10. Importance ranking of trace metals in sediment to explain the change in the dominant phylum abundance of bacterial community in different spatial units at the watershed scale.

Environment factors	Important ranking	Explanation (%)	F	*P*
Fe	1	40.0	22.7	**0.002**
Mn	2	24.2	10.9	**0.002**
Al	3	21.2	9.2	**0.002**
Sr	4	14.4	5.7	**0.010**
Cu	5	13.4	5.3	**0.016**
As	6	11.7	4.5	**0.028**
Cr	7	4.4	1.6	0.222
Ni	8	1.7	0.6	0.638
Zn	9	0.7	0.2	0.878

Significant results are shown in bold.

Table. S11. Importance ranking of trace metals in sediment to explain the change in the diversity of bacterial community in different spatial units at the watershed scale.

Environment factors	Important ranking	Explanation (%)	F	*P*
Fe	1	27.0	12.5	**0.002**
Mn	2	15.7	6.3	**0.008**
Sr	3	14.0	5.5	**0.036**
As	4	13.3	5.2	**0.018**
Al	5	12.7	4.9	**0.040**
Cu	6	6.8	2.5	0.110
Cr	7	0.8	0.3	0.586
Zn	8	0.7	0.2	0.724
Ni	9	0.3	0.1	0.836

Significant results are shown in bold.

Table. S12. Importance ranking of trace metals in sediment to explain the change in the metabolism abundance of bacterial community in different spatial units at the watershed scale.

Environment factors	Important ranking	Explanation (%)	F	*P*
Fe	1	26.5	12.3	**0.004**
Mn	2	16.3	9.4	**0.004**
As	3	11.3	7.9	**0.012**
Cr	4	5.5	4.2	**0.036**
Cu	5	3.5	2.9	0.100
Sr	6	1.0	0.8	0.360
Al	7	0.3	0.3	0.686
Zn	8	0.3	0.2	0.716
Ni	9	0.2	0.2	0.728

Significant results are shown in bold.

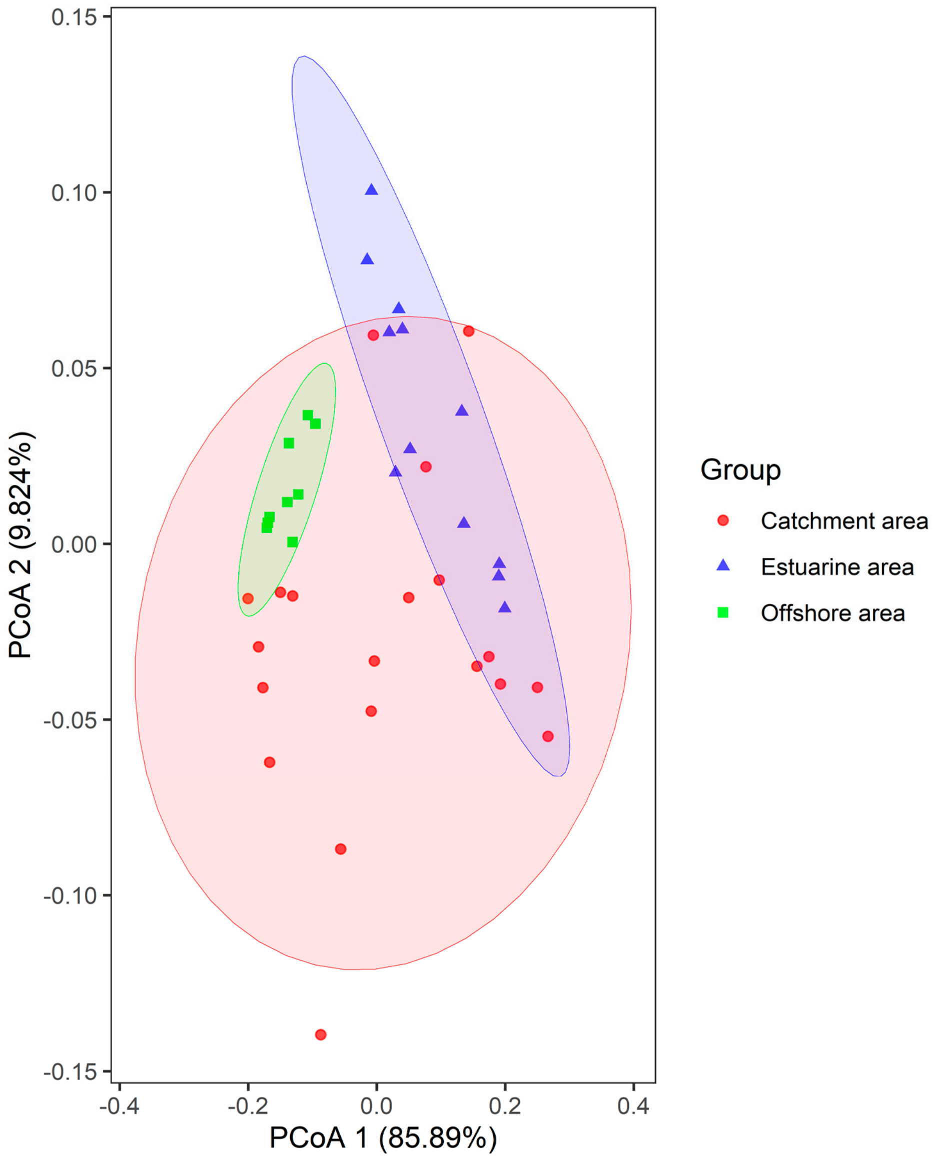

Fig. S1. Principal coordinates analysis (PCoA) of the variability between TMs contents in sediments from different spatial units at the watershed scale.

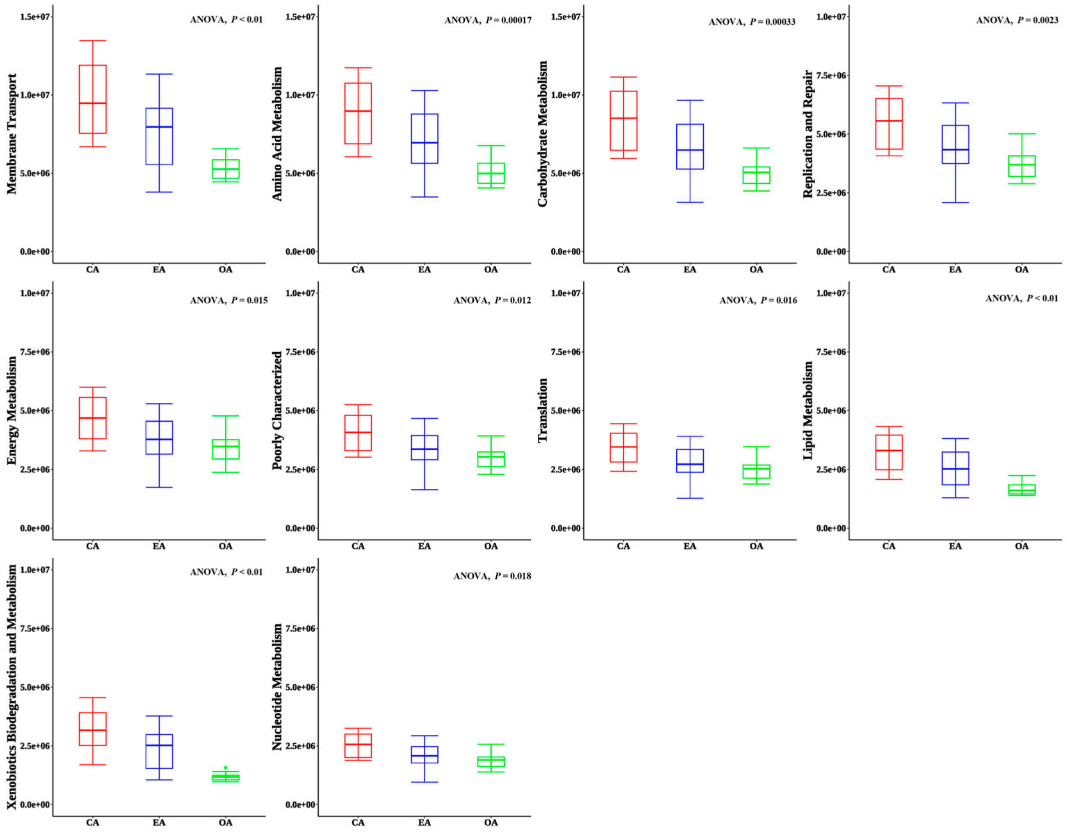

Fig.S2. Metabolic abundance of bacterial communities on KEGG database in different spatial. Among them, one-way ANOVA was used to test for variability in metabolic abundance at the watershed scale.

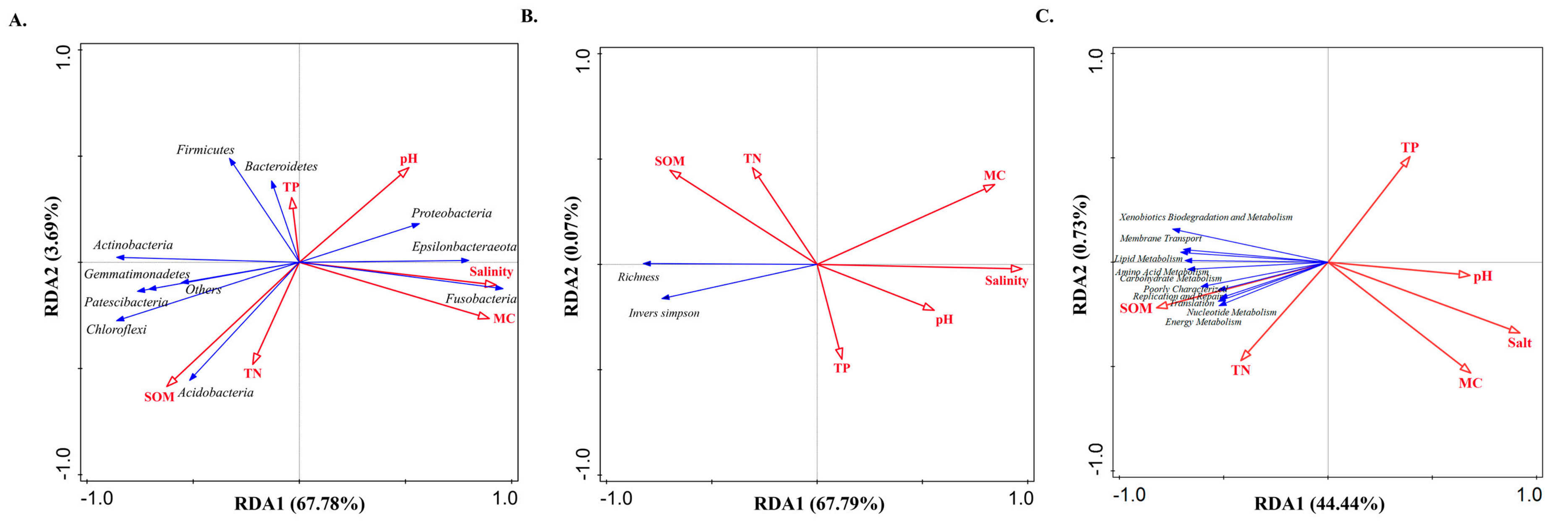

Fig. S3. Redundancy analysis (RDA) of environmental factors with bacterial community dynamics. A. effect of environmental factors on the dominant community abundance; B. effect of environmental factors on the diversity; C. effect of environmental factors on the metabolism abundance.
